# Transcriptome analysis of five ovarian stages reveals gonad maturation in female *Macrobrachium nipponense*

**DOI:** 10.1186/s12864-021-07737-5

**Published:** 2021-07-06

**Authors:** Yuning Zhang, Sufei Jiang, Hui Qiao, Yiwei Xiong, Hongtuo Fu, Wenyi Zhang, Yongsheng Gong, Shubo Jin, Yan Wu

**Affiliations:** 1 Wuxi Fisheries College, Nanjing Agricultural University, 214081 Wuxi, China; 2 Key Laboratory of Freshwater Fisheries and Germplasm Resources Utilization, Freshwater Fisheries Research Center, Ministry of Agriculture, Chinese Academy of Fishery Sciences, 214081 Wuxi, China

## Abstract

**Background:**

*Macrobrachium nipponense* is an economically important species of freshwater shrimp in China. Unlike other marine shrimps, the ovaries in adult female *M. nipponense* can mature rapidly and periodically during the reproductive period, but the resulting high stocking densities and environmental deterioration can negatively impact the harvest yield and economic benefits. To better understand ovary development in female *M. nipponense*, we performed systematic transcriptome sequencing of five different stages of ovarian maturation.

**Results:**

We obtained 255,966 Gb of high quality transcriptome data from 15 samples. Of the 105,082 unigenes that were selected, 30,878 were successfully annotated. From these unigenes, we identified 17 differentially expressed genes and identified three distinct gene expression patterns related to different biological processes. We found that cathepins, legumains, and cystatin were enriched in the lysosome pathway, and they are related to vitellogenin hydrolysis. Additionally, we found that myosin heavy chain 67 participated in oocyte excretion.

**Conclusions:**

We provide the first detailed transcriptome data relating to the ovarian maturation cycle in *M. nipponense*. Our results provide important reference information about the genomics, molecular biology, physiology, and population genetics of *M. nipponense* and other crustaceans. It is conducive to further solve the problem of *M. nipponense* rapid ovarian maturation from the aspects of energy supply and cell division.

**Supplementary Information:**

The online version contains supplementary material available at 10.1186/s12864-021-07737-5.

## Background

*Macrobrachium nipponense* (Crustacea; Decapoda; Palaemonidae) is widely distributed in China, Japan, Korea, Vietnam, and Myanmar [1, 2]. There is a huge market demand for this species, with high prices and a tight market, due to its taste and nutritional value. During the reproductive period, the ovaries of adult female *M. nipponense* mature rapidly and periodically, resulting in the production of huge numbers of offspring in the culture ponds and subsequent high stocking densities and environmental deterioration. This pattern of ovarian maturation has not been reported in other shrimp species and appears to be unique to *M. nipponense*. It differs markedly from the pathological sexual precocity demonstrated by crustacean species such as *Eriocheir sinensis* and *Macrobrachium rosenbergii* [3–5]. This reproductive phenomenon in *M. nipponense* results in a significant decline in the market specifications of females, ultimately affecting the whole harvest yield and causing economic losses. Thus, understanding the molecular mechanisms that regulate ovarian maturation in this species has dual significance in terms of production practices and scientific research.

As the ovary of *M. nipponense* matures, its size and colour undergo visible changes. From March to April every year, when water temperature increases, the winter-stagnant ovaries begin to develop. Development proceeds as follows: undeveloped stage (stage I: transparent, oocyte proliferation), developmental stage (stage II: khaki, original age of yolk), near maturity stage (stage III: light green, secondary yolk), mature stage (stage IV: dark green, yolk termination), and recession stage (stage V: dark gray). The surface of the ovary in stage I is uneven, and it is transparent with small brown spots. In stage II, the ovaries are khaki and slightly larger in volume, with a larger spot area, compared to stage I. The ovaries in stage III expand rapidly, increase significantly in volume, are light green in colour, and have a smooth surface. Ovaries in stage IV are the largest in size and dark green in colour, and distinct eggs can be seen through their walls. In stage V, ovaries in recession have been emptied of eggs, the size is close to that found at stage II, and the colour is opaque but darker than that of the developmental stage (。. S1).

To date, researchers have cloned, expressed, and studied the functions of ovarian maturation-related genes such as *GIH*, *GnRH*, *VG*, *VGR, Cathepsin*, and *HSP90* [6–10]. These genes also have been studied in species related to *M. nipponense*, such as *Macrobrachium rosenbergii* [11–13] and *Penaeus vannamei* [14, 15]. However, no key regulatory genes have been identified in *M. nipponense*, and the regulation of ovarian maturation remains unclear. No reference genome for *M. nipponense* is available to date, and ovarian-related research currently is based on transcriptome sequencing, which can dynamically reflect the levels of gene transcription and provide a molecular basis for biological research [16]. Only two transcriptome libraries suitable for ovarian research in *M. nipponense* have been reported. Wu et al. [17] constructed an ovarian cDNA library and Jiang et al. [18] compared normal and precocious sexually mature ovaries by transcriptome analysis of *M. nipponense*. However, limitations in detection technology meant that the amount of effective information available in the libraries was small. Moreover, Jiang et al. [18] distinguished between and compared sexually precocious and normal mature ovarian transcription groups; their results reflected differences between the ovarian-transcription groups under specific conditions but did not reveal the whole process of ovarian maturation. Thus, information about ovarian maturation is lacking for *M. nipponense*.

Transcriptomic characterizations of gonadal maturation in fish species such as *Coilia nasus* [19] and *Oreochromis niloticus* [20] and other organisms (fish, turtles, decapods) have been carried out [21–24]. These studies demonstrate the feasibility of obtaining transcriptional profiles of ovaries at different stages of maturation to identify the mechanisms that underlie *M. nipponense* gonadal maturation. In this study, we first obtained transcriptome data integrated from five stages of gonad maturation in female *M. nipponense*, then we used comparative transcriptomics to identify differentially expressed genes (DEGs) and annotated them using the gene ontology (GO) and Kyoto Encyclopedia of Genes and Genomes (KEGG) databases [25]. Cluster analysis illustrated different expression trends of DEGs involved in various biological processes, and the 17 DEGs identified revealed the genes and pathways involved in the internal regulation of ovarian development. These data are a valuable resource for elucidating the molecular mechanisms that underlie ovarian maturation in *M. nipponense*.

## Results

### Summary statistics of transcriptome sequencing and de novo assembly

**Fig. 1:**
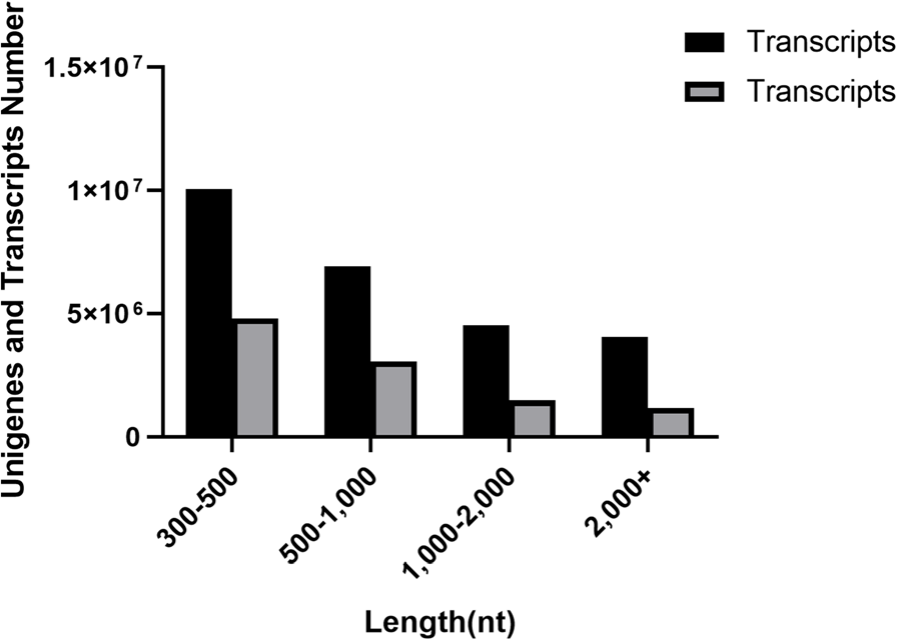
Length distribution of unigene. The abscissa represents different length ranges of unigenes, and the ordinate represents the number and proportion of unigenes in a certain length range

After filtering out low quality reads using the Illumina HiSeq 2500, we obtained the following data for the five stages of ovarian development: 72,858,556 clean reads for stage I; 75,022,388 for stage II; 68,107,104 for stage III; 81,419,654 for stage IV; and 66,749,538 for stage V (Table 1). All sequence reads were deposited in the National Center for Biotechnology Information (NCBI) Sequence Read Archive (accession SAMN11603268-SAMN11603282) under Bioproject PRJNA541783. Q20 values of all samples, as detected by FastQC26, were higher than 95 %, which indicated high quality of sequencing. Clean reads were pooled and assembled into nonredundant transcripts without reference genomes using Trinity software. In total, 255,966 transcripts and 105,082 unigenes were generated with average length of 1173.15 base pairs (bp) and 988.46 bp, respectively, with contig N50 values of 2001 and 1552. Figure 1 shows the distribution of all of the de novo assembled transcripts with different sizes.

**Table 1:** Quality control and data statistics for clean reads

Sample	Read Number	Base Number	Q20 (%)	GC (%)
T1-1	19,890,704	5,967,211,200	96.27	43.88
T1-2	26,650,589	7,995,176,700	97.3	42.33
T1-3	26,117,263	7,835,178,900	96.75	42.03
T2-1	24,548,501	7,364,550,300	96.37	41.98
T2-2	27,839,800	8,351,940,000	96.79	42.33
T2-3	22,634,087	6,790,226,100	96.49	42.41
T3-1	22,242,889	6,672,86,6700	96.03	42.13
T3-2	23,240,591	6,972,177,300	95.73	39.3
T3-3	22,623,624	6,787,087,200	95.95	41.17
T4-1	25,169,433	7,550,829,900	96.12	41.67
T4-2	36,997,530	11,099,259,000	96.51	40.33
T4-3	19,252,691	5,775,807,300	97.47	39.92
T5-1	19,972,636	5,991,790,800	96.56	41.74
T5-2	25,857,241	7,757,172,300	96.47	41.36
T5-3	20,919,661	6,275,898,300	96.61	41.38

### Assembly and functional annotation

In total, 30,878 unigenes with an E-value of < 10^− 5^ were annotated into seven databases to identify putative functions using Blastx. Among them, 6817, 5927, 6533, 14,393, 14,857, 17,393, and 30,305 unigenes were identified in the COG, GO, KEGG, KOG, Pfam, Swissprot, and NR databases, respectively (Table S1).

**Fig. 2:**
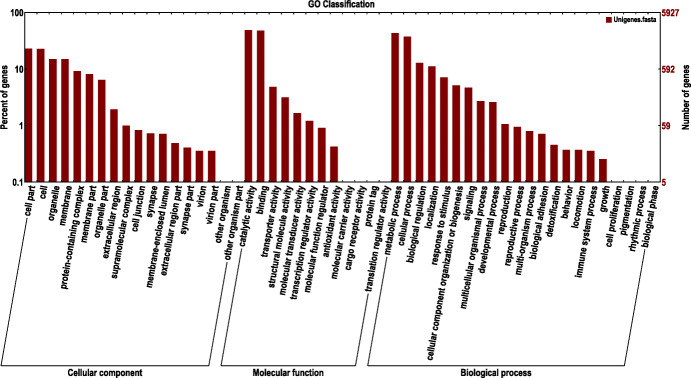
GO classification of unigenes; The abscissa is the second level term under the three categories of GO. The ordinate represents the number of genes annotated to the term and the percentage of all genes

**Fig. 3:**
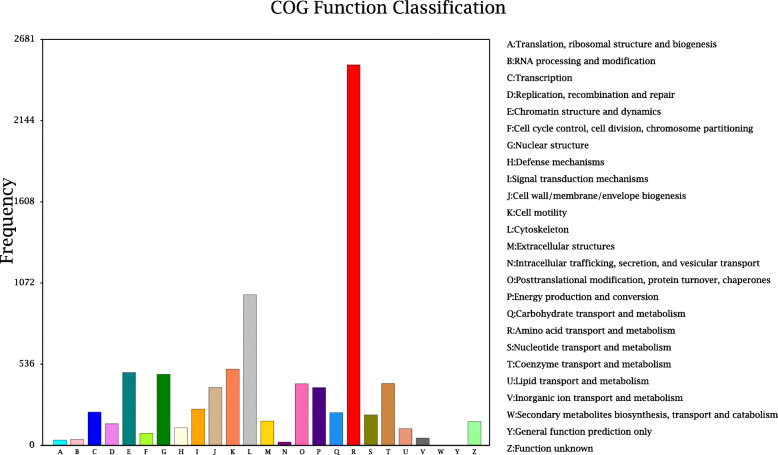
COG classification statistics of unigenes. The abscissa is the classification content of COG, and the ordinate is the number of genes

**Fig. 4:**
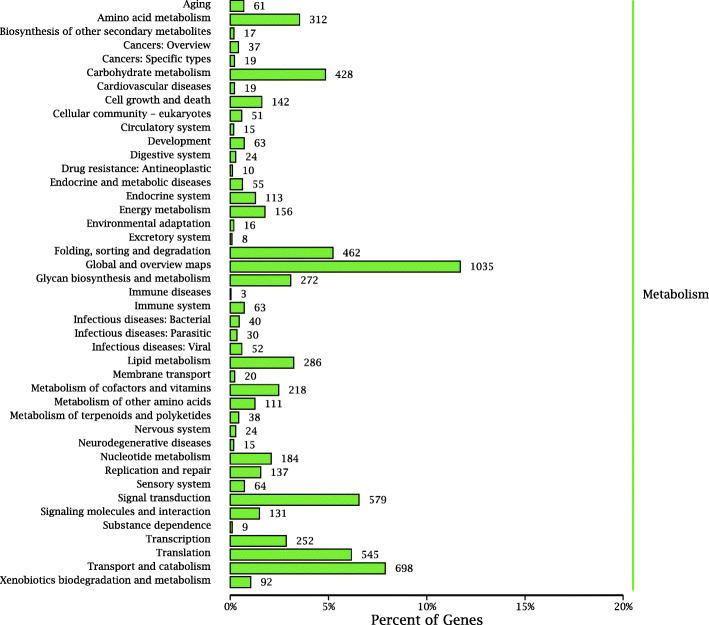
KEGG pathway distribution of unigenes. The ordinate (left) is the name of KEGG secondary metabolic pathway, the ordinate (right) is the name of KEGG primary metabolic pathway, and the abscissa is the number of genes annotated to the pathway and the proportion of the number of genes annotated to the total number of genes annotated

Gene products were clustered by the GO and COG databases to describe their functional attributes. GO analysis grouped 5,927 unigenes into three major functional categories: biological process (38.16 %); molecular function (31.28 %); and cellular component (30.56 %). The most annotated functional categories were catalytic activity (14.02 %), binding (13.70 %), and metabolic process (12.45 %) (Fig. 2). Based on sequence homology, 6,817 unigenes were classified into 25 functional categories in the COG database, with amino acid transport and metabolism annotated to the largest number of unigenes (up to 2,034), followed by cytoskeleton and cell motility (Fig. 3). KEGG analysis was applied to identify the biological pathways that are related to the unigenes. In total, 6,533 unigenes were highly matched to known genes and assigned to 305 signaling pathways. The top five pathways were global and overview maps (1,035, 15.84 %), transport and catabolism (698, 10.68 %), signal transduction (579, 8.86 %), translation (545, 8.34 %), and folding, sorting and degradation (462, 7.07 %) (Fig. 4).

### Identification and functional analysis of DEGs

Using the criteria of |log2Ratio| ≥ 1 and FDR < 0.01, we found 4655 DEGs in the pairwise comparison of ovaries from the five developmental stages (2,075 up-regulated, 2,580 down-regulated). When we focused on the process of ovarian maturation, we identified 262 DEGs between stages I and II, with 79 up-regulated and 183 down-regulated significantly. For stages II to III, we found 232 up-regulated and 117 down-regulated genes, and for stages III to IV and stages IV to V, the numbers were 272 (65 up-regulated, 207 down-regulated) and 712 (476 up-regulated, 236 down-regulated) (Table 2, Table S2).

**Table 2:** DEG statistics for comparison groups

	O I vs. O II	O II vs. O III	O III vs. O IV	O IV vs. O V
Up-regulated	79	232	65	476
Down-regulated	183	117	207	236

We used GO and KEGG enrichment analysis to determine the potential functional expressions of DEGs. For the four comparisons (stages I to II, II to III, III to IV, and IV to V), we found 45, 158, 23, and 106 DEGs, respectively, that were annotated into 15, 48, 10 and 38 GO categories. Figure S2 shows the functional classification of the 10 most significant nodes in each database (biological process, cellular component, and molecular function). The most pathways occurred in the comparison of stage II vs. III, with 33 DEGs enriched in 35 pathways. Among them, seven pathways (e.g., lysosome, autophagy-animal, and amino sugar and nucleotide sugar metabolism) were significantly different between the two stages (q-value < 1). The comparison of stages III and IV had the lowest number of enriched DEGs, with only two DEGs annotated in four pathways. Three of the pathways (synthesis and degradation of ketone bodies, butanoate metabolism, and neuroactive ligand-receptor interaction) were significantly different (q-value < 1). There were 12 and 13 DEGs mapped to 22 and 18 pathways in the comparisons of stage I vs. II and stage IV vs. V. Glutathione metabolism, metabolism of xenobiotics by cytochrome P450, and four other pathways were significantly different between stages I and II. IN the stage IV vs. V comparison, tight junction, amoebiasis, and three other pathways differed significantly (Fig. S3).

### Clustering of DEGs revealed distinct expression patterns

**Fig. 5:**
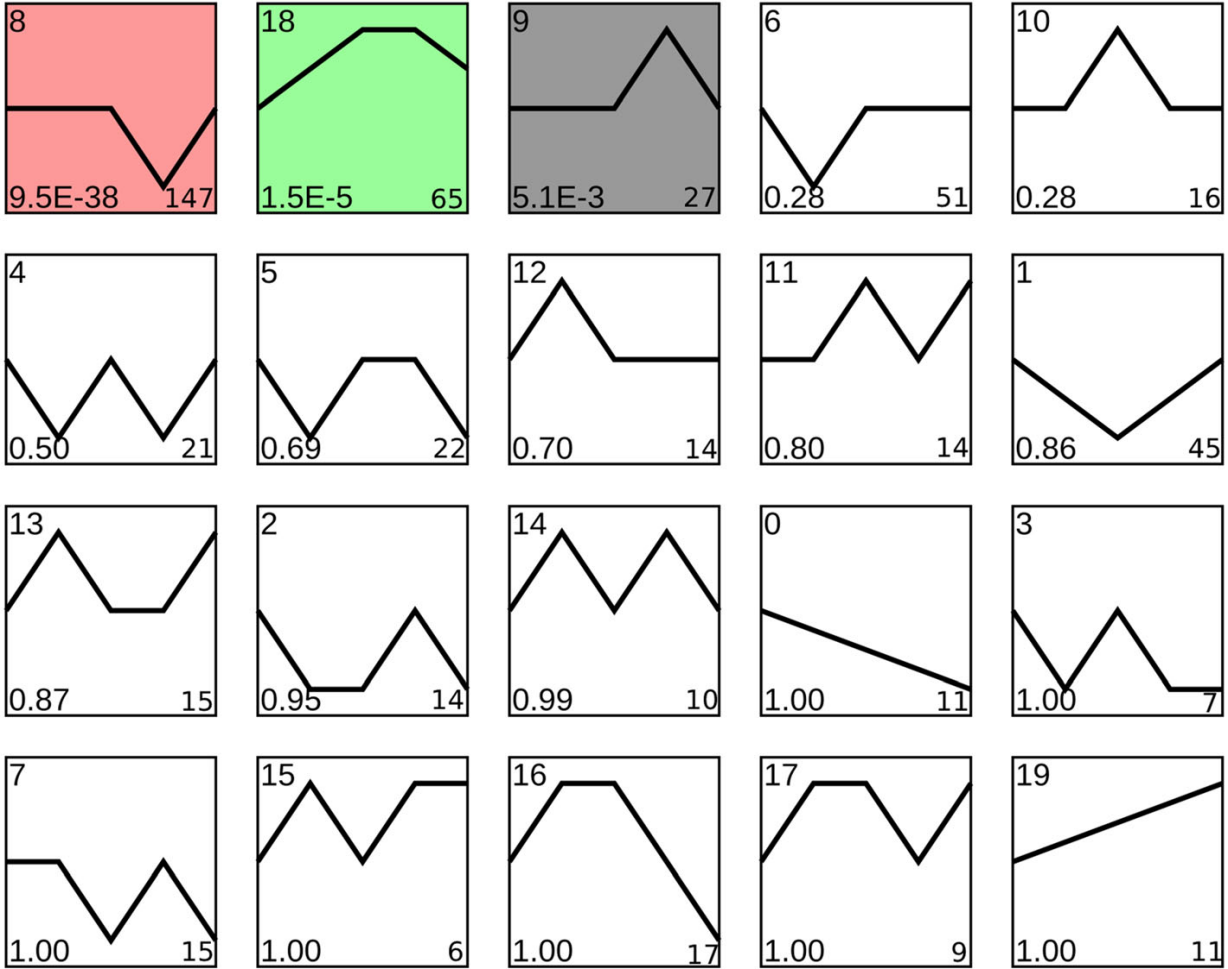
Significant expression patterns of DEGs were found for three of the 20 profiles (in color). The lower left of each profile is the P-value, and the lower right is the number of DEGs

To evaluate the variation of expression of these genes at multiple time points during the process of ovarian maturation, we used series test of cluster (STC) analysis and found three clusters with significant expression patterns (*P* < 0.05) (Fig. 5). We identified 147 DEGs with the same pattern (profile 8), which was upward from stage III to IV and downward from stage IV to V, as well as 27 DEGs that showed the opposite pattern (profile 9). The third significant expression pattern (profile 18) shown by 65 DEGs was up-regulation from stage I to III and down-regulation from stage IV to V. Profile 8 shows that 63 DEGs were classified into five biological processes in COG (replication, recombination and repair (56), general function prediction only (4), posttranslational modification, protein turnover, chaperones (1), cell wall/membrane/envelope biogenesis (1), and cell motility (1)) (Table S3). Only two DEGs were annotated to general function prediction only and replication, recombination and repair, respectively, in profile 9 (Table S4). In profile 18, 20 % of the DEGs (13) were enriched to six biological processes, and most were in the general function prediction only category (Table S5).

### Screening and expression of genes related to ovary maturation

**Fig. 6:**
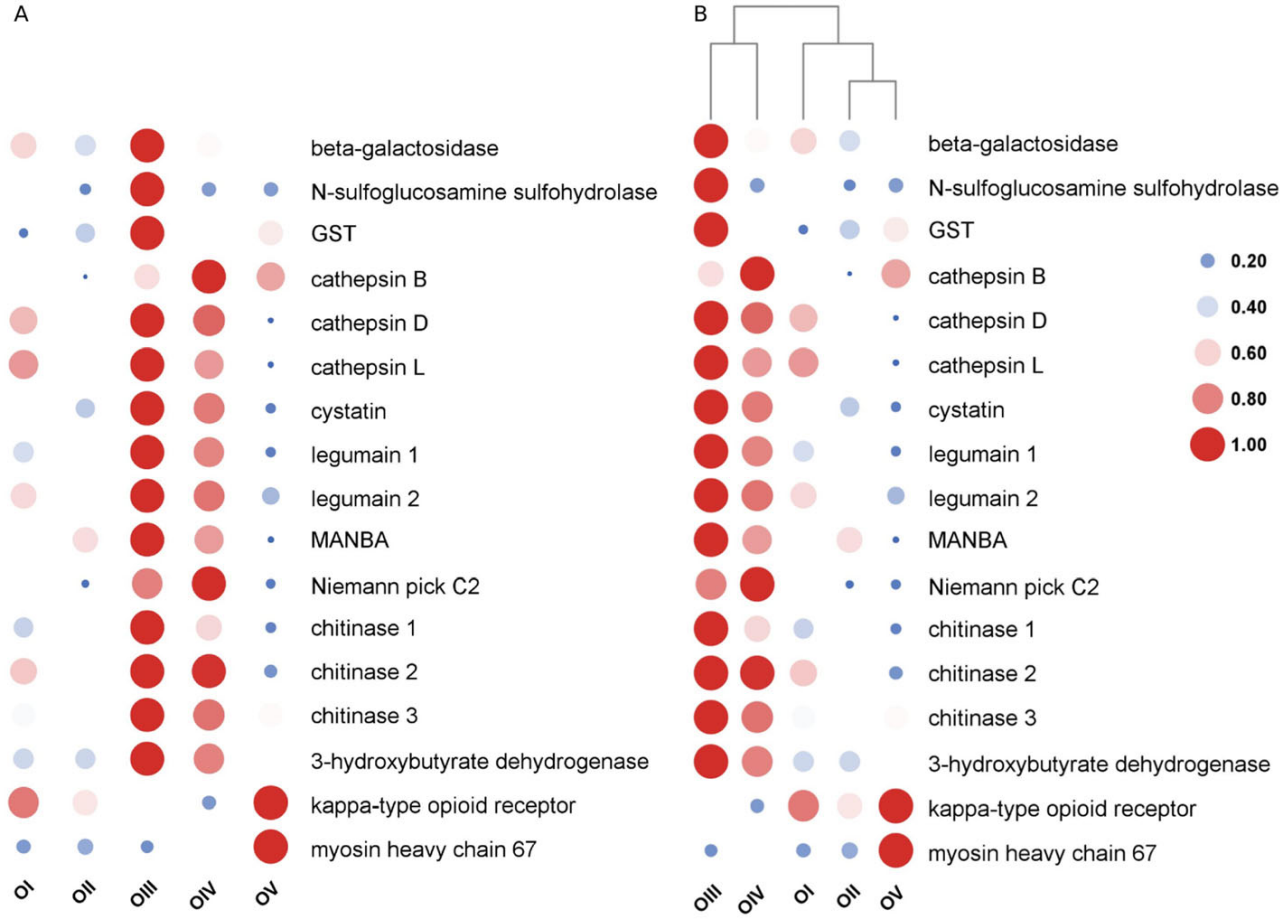
Heat map of genes related to ovary maturation. A: Quantification analysis of ovarian development related genes at the five different development stages. B: Clustering of the genes during the five different development stages

**Fig. 7:**
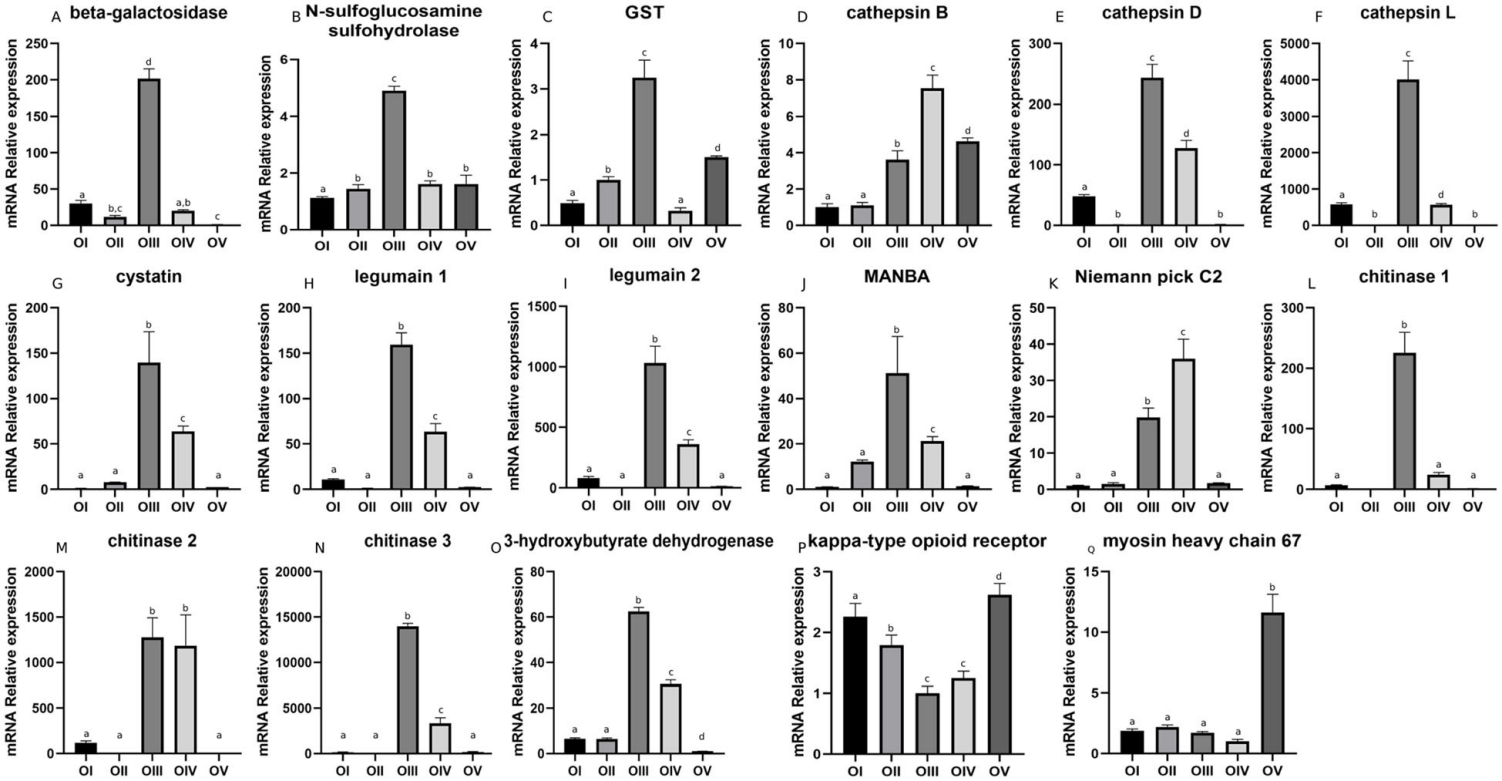
The qRT-PCR results for ovarian development related genes. O I, Undeveloped stage; O II, developed stage; O II, near mature stage; O IV, mature stage; O V, declining stage. A: beta-galactosidase, B: N-sulfoglucosamine sulfohydrolase, C: GST, D: cathepsin B, E: cathepsin D, F: cathepsin L, G: cystatin, H: legumain 1, I: legumain 2, J: MANBA, K: Niemann-Pick C2, L: chitinase 1, M: chitinase 2, N: chitinase 3, O: 3-hydroxybutyrate dehydrogenase, P: kappa-type opioid receptor, Q: myosin heavy chain 67. The same letter indicates no significant difference between the time points (*P* < 0.05; mean ± SE, *n* = 3)

Based on the analysis of DEGs, we screened 17 genes related to ovarian maturation by combining the q-value of the DEG enrichment pathways in the comparison groups I vs. II, II vs. III, III vs. IV, and IV vs. V. Twelve genes (beta-galactosidase, N-sulfoglucosamine sulfohydrolase, glutathione S-transferase (GST), cathepsin D, cathepsin L, cystatin, legumain 1, legumain 2, MANBA, chitinase 1, chitinase 3, and 3-hydroxybutyrate dehydrogenase) displayed similar expression patterns, which increased and then peaked at stage III and then decreased. The expression pattern of the kappa-type opioid receptor gene showed the opposite pattern, with the lowest expression at stage III followed by a gradual increase. The expression of cathepsin B and Niemann-Pick C2 genes increased with ovary development, peaking at stage IV and then decreasing. The T chitinase 2 gene was highly expressed at stage III and maintained a high-level during stage IV. Myosin heavy chain 67 expression was low in the first four stages of ovary maturation then increased at stage V (Fig. 6 A, Fig. 7). The heat map of these genes showed the differential expression patterns and how they clustered (Fig. 6B). Figure 7 shows great differences in gene expression patterns between stages III–IV and the other development stages.

## Discussion

In this study, we conducted transcriptome analysis for the five developmental stages of the *M. nipponense* ovary in order to identify the genes related to ovary development. Understanding the internal transcriptional regulation changes that occur during the physiological process of ovary development will help us proactively solve various production problems caused by ovary maturation in this species.

The quality control analysis of clean reads showed that the base recognition in this study was reliable and that the error rate was low, thus they met the requirements for sequence assembly. After annotation, enrichment, and functional classification, we identified a large number of pathways and categories related to ovarian maturation. These predicted biological processes and pathways helped explain the relationship among genes and the regulatory mechanisms that were at work. Comparison of development stages III and IV revealed the fewest DEGs (23), which were annotated to three significant pathways. These results suggested that these two periods are relatively similar. Based on identification of DEGs combined with our knowledge of the physiological process of ovarian development, stages I, II, and V were referred to as the oogenesis period and stages III and IV were referred as the vitellogenesis period. These two periods have different characteristic metabolites [26]. Thus, both metabolomics and transcriptomics data showed that stages III and IV were different from the other stages, and the biological processes at work during these two stages require more intensive study.

Physiological processes that occur during stages II and III differed greatly, which likely explains the large difference in the transcriptome sequencing results for this comparison. Lysosome was the most significant pathway and contained the largest number of DEGs (7). Lysosome is involved in various cellular processes, such as cholesterol homeostasis, autophagy, membrane repair, bone and tissue remodeling, pathogen defense, cell signaling, and death [27, 28]. As the central digestive organ of cells, various macromolecules are sent to the lysosome for degradation. In recent years, researchers have focused on the potential relationship between lysosomes and ovarian development. For example, Carnevali et al. proposed that lysosomes play an important part in the degradation of vitellogenin (VG) [29]. VG is the precursor of vitellin (Vn), which is an important source of nutrients for embryonic and gonadal development, and VG is an important factor affecting ovarian development. In addition, salmon gonadal maturation was found to be related to proteolysis [30], which suggests that lysosomes are involved in energy redistribution. Therefore, it is widely accepted that lysosomes are related to gonadal development of most fishes. The activation of lysosomes is accompanied by energy directed to reproduction. Therefore, we hypothesize that lysosomes are related to the hydrolysis of VG and energy demand during *M. nipponense* ovarian maturation.

In our study, the DEGs enriched in the lysosome pathway were cathepsin B, D, L, legumain, Niemann-Pick C2, MANBA, and cystatin, and qRT-PCR analysis showed that their expression levels were significantly increased from stage II to III (the period of oogenesis to vitellogenesis). Previous studies in fish and insects showed that cathepsin D is the enzyme that allows endocytic VG to produce Vn. For example, high cathepsin D activity was correlated with high cathepsin B activity, indicating that cathepsin B may participate in VG digestion by activating cathepsin D [29]. Zhao et al. cloned the full-length cathepsin L gene and found that it was highly expressed during the later stage of vitellogenesis in the *M. nipponense* ovary and in the hepatopancreas, and then expression decreased with development of the embryo [9]. Thus, cathepsin L affects production and hydrolysis of Vn during ovarian maturation and embryo development.

Our comparison of stage II vs. III reflected the changes that occur from vitellogenesis to oogenesis. During this period, the expression of Mn-VG was up-regulated [7], which is consistent with the changes observed for cathepsin B, D and L. This indicated that cathepsins play a role in ovarian development as key factors that affect the process of VG hydrolysis to promote maturation. Legumain is a cysteine endopeptidase with strict specificity to the hydrolysis of asparagine bonds, which has been reported in shrimp immune and stress research [31]. It also has an effect on intestinal digestion [32]. However, little is known about this gene’s role in the field of gonadal development, and we can only suggest that legumain may be involved in the digestion of VG and ovarian development. Studies of cystatin indicated it is not only related to the development of the male gonad but that it also plays an important role in the process of oogenesis, and it can inhibit cathepsin, especially cathepsin B [33–35]. Cystatin can inhibit the activity of legumain to protect cells from the adverse effects of proteolysis. Based on our results, we suggested that the positive feedback of cystatin and other genes cooperatively protected the progress of ovarian maturation. Niemann-Pick C2 can specifically bind with cholesterol and plays an important role in regulating cholesterol homeostasis in normal cells [36, 37]. Cholesterol is accumulated through endogenous production and exogenous transport. These results suggested that Niemann-Pick C2 may be involved in the transport and distribution of cholesterol during ovarian maturation. MANB belongs to the glycohydrolase family, which has been found in the proteome of mammalian sperm epididymal maturation, but there are no data about its presence or role in the ovary. Overall, we do not have enough information to draw a conclusion about the role of these genes in ovarian maturation, and functional studies of these genes is required.

In the comparison of stage IV vs. V, myosin heavy chain 67 was a notable DEG enriched in the top three significant KEGG pathways. Myosins, which are best known for their roles in muscle contraction, provide the motor function for diverse movements such as cytokinesis, phagocytosis, and muscle contraction [38]. Kelley and Cram recently reported that myosins contract in a coordinated manner to regulate oocyte entry and exit of the fertilized embryo into the uterus [39]. In our study, the expression of myosin heavy chain 67 was significantly up-regulated only at stage V, probably because oocytes were expelled from the ovary at this stage.

To identify the most representative gene group active during the process, we conducted STC analysis of the DEGs. In profile 8, replication, recombination and repair were the most enriched GO functional pathway, with 56 DEGs, and this pathway participated in the biological process of cell division. Whether cells were undergoing mitosis or meiosis, the interphase and prophase stages involved DNA replication, recombination, and repair. Earlier studies of the transition from stage I to III reported abundant oogonium proliferation through mitosis followed by the first meiosis to become secondary oocytes [40–42]. In the interim, genes enriched for replication, recombination, and repair remain at a high level of expression. Histological results showed the presence of numerous mature oocytes in stage IV of the ovary [40–42]. During the period of ovarian maturation (stages III to IV), no DNA synthesis occurred, thus the expression of related genes decreased. After oocyte maturation, the ovary prepared for the next cycle of oogenesis and oogonium proliferation, and expression of related genes increased. These results revealed that oocyte maturation was an important biological process during ovarian maturation and that many related genes exhibited a regular expression pattern. At present, these genes lack annotation information and require more research.

Most DEGs in profile 18 were annotated to general function prediction only, with the pattern of up-regulation in stages I to III and down-regulation in stages IV and V. These genes included the feminization-1c (fem-1c) gene. Fem-1 genes play an essential role in the sex-determination/differentiation pathway in *Caenorhabditis elegans*. Ma et al. cloned a fem-1 homolog from *M. nipponense* and found that RNA and protein were exclusively expressed in the ovary in adult prawns. This finding suggested that Mnfem-1 could have roles in prawn ovarian development and sex determination/differentiation [43], which is consistent with the effect of fem-1c on zebrafish [44]. Moreover, fem-1c in the mussel *Hyriopsis cumingii* was found to be involved in female gonad differentiation and to participate in egg development [45]. Therefore, fem-1c might play a role in *M. nipponense* ovary maturation. We also found that some zinc finger proteins with unknown functions were enriched in this profile. Some zinc finger proteins acted as transcription factors to regulate transcription of target genes, and some specifically mediated protein interactions. Some zinc finger proteins also bind to RNA and play a post transcriptional regulatory role [46]. Due to the lack of annotation information and related research, it is not clear which genes are involved in the transcription and modification of these zinc finger proteins, but we speculate that these genes are closely related to ovarian maturation.

## Conclusions

Results of this study provided comprehensive data about the ovarian maturation transcriptome in *M. nipponense*. We obtained 30,878 unigenes, of which 4,655 were found to be differentially expressed in comparisons of the five development stages of the ovary. We identified 17 DEGs that may be related to ovary maturation in significant pathways identified in the comparisons. Among them, cathepins, legumains, and cystatin were enriched in the lysosome pathway, and they are known to protect the progress of VG hydrolysis. Additionally, myosin heavy chain 67 participated in oocyte excretion. Our results highlighted differences between stages III-IV and the other stages of ovary development. The reason of the difference may due to the biological processes happened in each period. Our data provided new information about the regulation of ovarian maturation in *M. nipponense* and may be helpful for solving the problem of rapid *M. nipponense* development in the aquaculture industry. However, the relationship between other regulatory genes and ovarian maturation requires further study.

## Methods

### Tissue preparation and RNA isolation

We obtained healthy female *M. nipponense* (2.75 ± 1.45 g) from the Freshwater Fisheries Research Center of the China Academy of Fishery Sciences (Wuxi, China) (120°13’44′′E, 31°28′22′′N). Shrimp were cultured at 100 individuals/m^2^, and the feeding conditions followed Sun et al. [47]. According to previous studies [48], five different stages of ovarian maturation were distinguished by colour (see Fig. S1) and immediately stored in liquid nitrogen at -190 °C. Tissues were collected from five individuals at each stage and homogenized with TRIzol reagent (Autolab Tech, Beijing, China) to extract total RNA. The RNA concentration was detected using a Qubit RNA Kit with Qubit 2.0 Fluorometer (Life Technologies, Carlsbad, CA, USA), and its purity was detected using a Nanodrop 2000 spectrophotometer (Thermo Scientific, Waltham, MA, USA). The integrity of the RNA was assessed using an RNA Nano 6000 detection kit (2100 Bioanalyzer System; Agilent Technologies, Santa Clara, CA, USA).

### Library construction and sequencing

A sequencing library was prepared with 3 µg of RNA from each sample using a NEBNext Ultra RNA Library Prep Kit (Illumina, San Diego, CA, USA) according to the manufacturer’s instructions. The RNA was purified and broken into small random fragments using poly-T oligo attached magnetic beads (Life Technologies, Carlsbad, CA, USA). Double-stranded DNA was synthesized using a TruSeq™ Stranded mRNA Prep Kit (Illumina). The DNA fragments in the library with a length of 150–200 bp were screened and purified using an Ampure XP system (Beckman Coulter, Beverly, MA, USA). The purified double-stranded cDNA (size selection and connection) was incubated with 3 ml of USER enzymes (NEB, Ipswich, MA, USA) at 37 °C for 15 min and then cultured at 95 °C for 5 min. Polymerase chain reaction (PCR) was performed with Phusion High-Fidelity DNA polymerase, universal PCR primers, and index (X) Primer, and the products were then purified using the Ampure XP system. The composite samples were paired and sequenced using a HiSeq™ 25,000 for 2 × 100 bp according to the manufacturer’s instructions. The PE reading of each lane was about 150 m (*n* = 3).

### Assembly and dataset annotation

An Illumina HiSeq high-throughput sequencing platform based on sequencing by synthesis technology can produce a large quantity of high-quality raw data. FastQC tools were used to truncate adapter and primer sequences and remove reads with N > 10 % (where N indicates inability to determine base information) and those with quality (Q) < 5 for > 50 % of reads. Trinity (http://trinityrnaseq.sourceforge.net/) was used to assemble reads from scratch according to parametric transcription group. The minimum contig length was 300 and K-mer was 27.

We annotated the final set of unigenes comprehensively using BLAST software (http://blast.ncbi.nlm.nih.gov/Blast.cgi) to compare unigene sequences with the NR (ftp://ftp.ncbi.nih.gov/blast/db/), Swiss-Prot (http://www.uniprot.org/), GO (http://www.geneontology.org/), COG (http://www.ncbi.nlm.nih.gov/COG/), KOG (http://www.ncbi.nlm.nih.gov/COG/), and KEGG (http://www.genome.jp/kegg/) databases (E-value ≤ 10^− 5^). After predicting the amino acid sequences of the unigenes, we compared them with the Pfam database (http://pfam.xfam.org/) using HMMER software (http://hmmer.janelia.org/).

### DEG analysis and quantitative analysis of ovarian maturation related gene expression at different ovarian developmental stages

We used DESeq2 to analyze the differential expression between the sample groups, and 10 pairwise comparative DEG sets of ovaries in the five stages were obtained. The Benjamini-Hochberg correction method was used to correct the significance of the p-value of the original test hypothesis to obtain the false discovery rate (FDR) [49], and fold-change was used to present the expression ratio between the comparison groups. |log_2_(fold change)| ≥ 1 and FDR < 0.05 were used as screening criteria to define DEGs. The GO, COG, and KEGG annotation methods were similar to those mentioned above in terms of unigene annotation, and DEG pathway enrichment analysis identified significantly enriched pathways based on a q-value < 0.05.

We validated the DEGs by qRT-PCR to evaluate the sequencing and data analysis. Total RNA was extracted from ovaries (~ 100 mg) with 1 mL TRIzol reagent (TaKaRa, Japan), and first-strand cDNA was synthesized using a Reverse Transcriptase M-MLV Kit (TaKaRa). The qRT-PCR was performed using a Bio-Rad iCycler iQ5 real-time PCR system (Hercules, CA, USA), with eukaryotic translation initiation factor 5 A as the reference gene [50]. The primers used are shown in Table S6. The reaction was amplified with 35 cycles at 94 °C for 30 s, 50 °C for 30 s, and 72 °C for 1 min, followed by 10 min incubation at 72 °C as a final extension step [51]. Four replicates were run for each sample, and three controls were used for each reaction: nuclease-free water; primer-free water; and template-free water. The fluorescence curve and data were recorded automatically by the system. The dissociation curves of the amplified products were analyzed at the end of each PCR. The mRNA expression levels were determined using the 2^−ΔΔCT^ method [52].

## Supplementary Information

**Additional file 1:**

**Additional file 2:**

**Additional file 3:**

**Additional file 4:**

## Data Availability

The dataset supporting the conclusions of this article is available in the NCBI Sequence Read Archive (accession SAMN11603268-SAMN11603282) under Bioproject PRJNA541783.
