# Supplementary figures and images for: Transcriptome analysis of five ovarian stages reveals gonad maturation in female Macrobrachium nipponense
Source: BMC Genomics. 2021 Jul 6;22:510. doi: 10.1186/s12864-021-07737-5 (PMC8262026; doi:10.1186/s12864-021-07737-5)

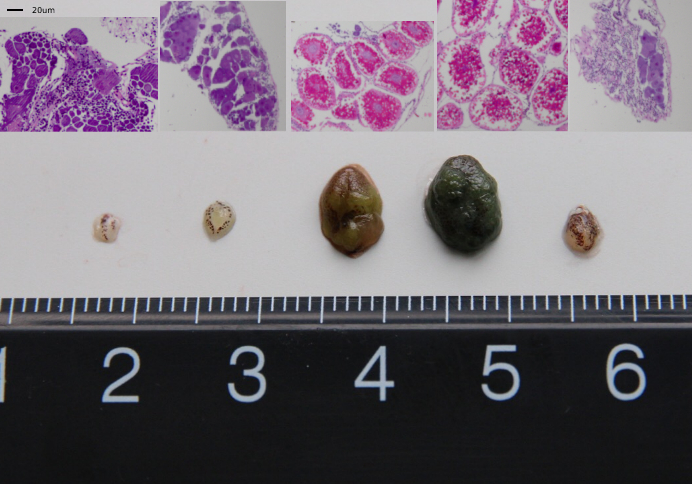

Supplement: Supplementary file 1 — Additional file 1: [file 12864_2021_7737_MOESM1_ESM.jpg]

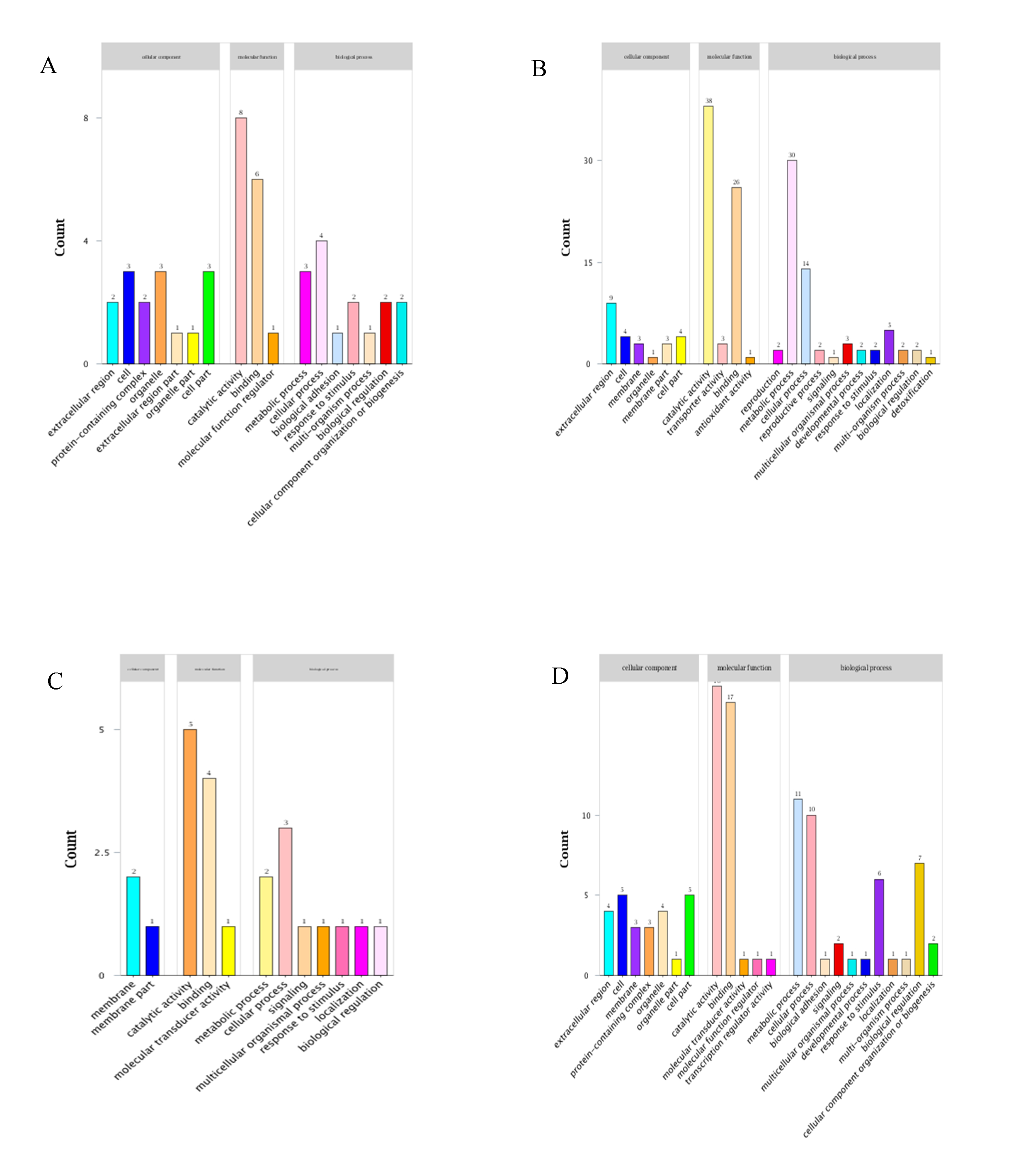

Supplement: Supplementary file 2 — Additional file 2: [file 12864_2021_7737_MOESM2_ESM.jpg]

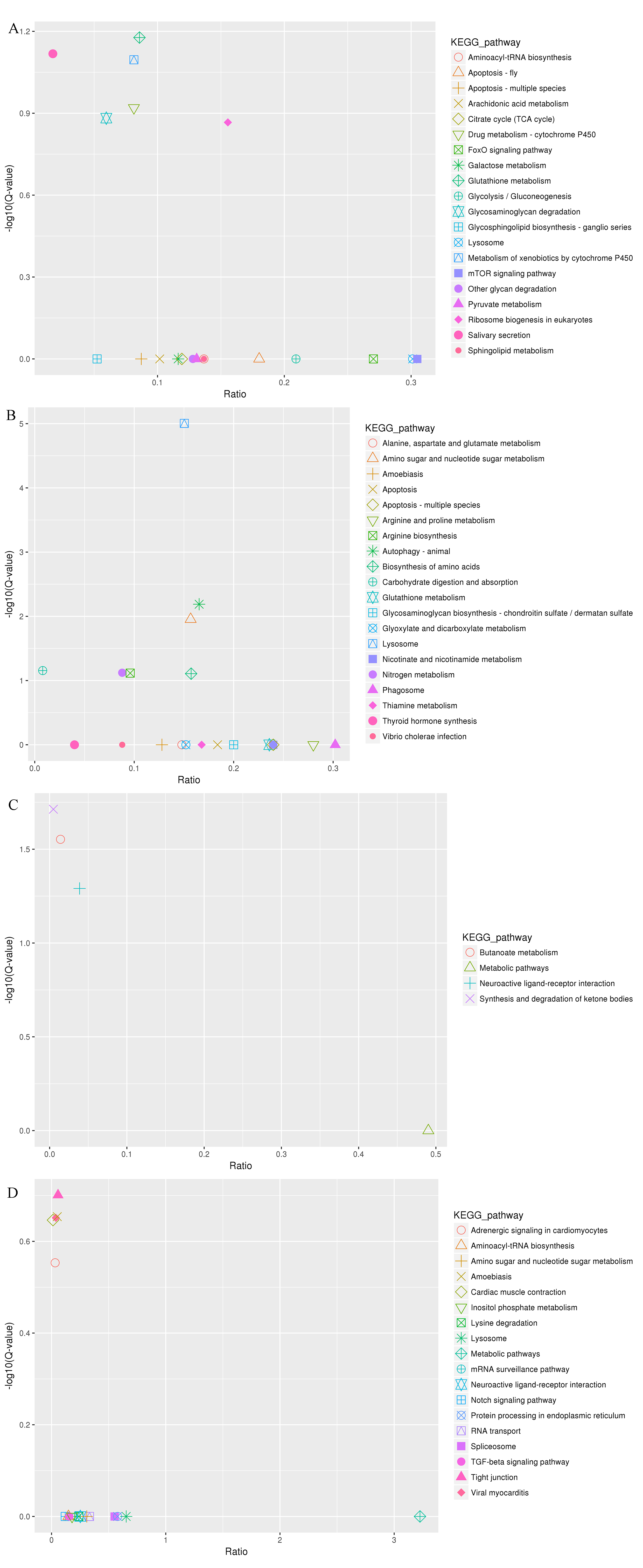

Supplement: Supplementary file 3 — Additional file 3: [file 12864_2021_7737_MOESM3_ESM.jpg]
